# rAAV-Mediated Cochlear Gene Therapy: Prospects and Challenges for Clinical Application

**DOI:** 10.3390/jcm9020589

**Published:** 2020-02-21

**Authors:** Fabian Blanc, Michel Mondain, Alexis-Pierre Bemelmans, Corentin Affortit, Jean-Luc Puel, Jing Wang

**Affiliations:** 1 INSERM - UMR 1051, Institut des Neurosciences de Montpellier, Hôpital Saint Eloi - Bâtiment INM, 80, rue Augustin Fliche - BP 74103, 34091 Montpellier, France; m-mondain@chu-montpellier.fr (M.M.); corentin.affortit@inserm.fr (C.A.); jean-luc.puel@inserm.fr (J.-L.P.); 2 Université de Montpellier, 163 rue Auguste Broussonnet, 34090 Montpellier, France; 3 CHRU Montpellier—Centre Hospitalier Régional Universitaire, 34090 Montpellier, France; 4 Molecular Imaging Research Center, Institut de Biologie François Jacob, Direction de la Recherche Fondamentale, CEA, 92265 Fontenay-aux-Roses, France; alexis.bemelmans@cea.fr; 5 Université Paris-Saclay, CEA, CNRS, UMR9199 Neurodegenerative Diseases Laboratory, 92265 Fontenay-aux-Roses, France

**Keywords:** rAAV, cochlear gene therapy, clinical application, genetic deafness, routes of delivery, serotypes, targets gene addition, RNAi, gene editing

## Abstract

Over the last decade, pioneering molecular gene therapy for inner-ear disorders have achieved experimental hearing improvements after a single local or systemic injection of adeno-associated, virus-derived vectors (rAAV for recombinant AAV) encoding an extra copy of a normal gene, or ribozymes used to modify a genome. These results hold promise for treating congenital or later-onset hearing loss resulting from monogenic disorders with gene therapy approaches in patients. In this review, we summarize the current state of rAAV-mediated inner-ear gene therapies including the choice of vectors and delivery routes, and discuss the prospects and obstacles for the future development of efficient clinical rAAV-mediated cochlear gene medicine therapy.

## 1. Introduction

Sensorineural hearing loss (SNHL) is one of the most prevalent sensory deficits in both childhood and adulthood, which affects approximately 466 million people worldwide and more than half of the population over 60 years of age. This is estimated to rise to more than 900 million people by 2050 [1]. SNHL is characterized by the degeneration of mechanosensory hair cells and the primary auditory neurons with their synaptic connection to the hair cells in the cochlea [2], which results in permanent hearing loss. SNHL is an etiologically heterogeneous disorder caused by environmental (e.g., ototoxic drugs, noise) and intrinsic causes (e.g., aging, genetic factors). It is estimated that about 80% of SNHL cases have a genetic cause, and only 20% have an environmental cause [3].

Clinically, genetic deafness resulting from monogenic disorders can be either congenital or late-onset [3]. Hearing loss can be associated with abnormalities in other parts of the body (called syndromic deafness) or without other signs and symptoms (non-syndromic). Specific genetic variants also contribute to the susceptibility of the individual to congenital, progressive, noise-induced and ototoxic drug-induced, and age-related, hearing loss [4].

In the past 20 years, significant progress has been made in our knowledge of the pathogenetic mechanisms of genetic or environmental deafness. Unfortunately, to date, there is still no cure for deafness. Some hearing rehabilitation is possible through hearing devices that can amplify sound, either by using conventional hearing aids in the case of mild or moderate deafness, or surgically-placed cochlear implants for severe bilateral deafness. The advantage of the latter is that it can bypass non-functional sensory hair cells by directly stimulating primary auditory neurons. However, despite the advances in hearing-aid and cochlear-implant technologies, the quality of perceived sound still cannot match that of the normal ear. Impaired speech perception in noisy environments [5] and musical sound perception [6] are the biggest hurdles faced by hearing-aid and cochlear-implant users.

Gene therapy is an experimental technique to introduce genetic materials into cells to prevent or treat a wide range of diseases. The advantage of this technique is the possibility of giving a person who was born with a genetic disease the chance of having a healthy life [7]. In the inner ear, recent results hold promise for the treatment of congenital or later-onset hearing loss and restoring hearing in monogenic disorders in patients [8,9,10]. Different factors, e.g., vector types, delivery routes, and regulatory elements, may impact both the inner-ear cell types targeted as well as transgene expression. In addition, compared to other target organs for gene therapy, there are several obstacles resulting from the unique anatomy of the inner ear. The translation of modern gene therapy into clinical practice is hampered by the “delivery” challenge.

In this review, we summarize and discuss recent advances in inner-ear gene transfer technologies aiming at restoring or protecting hearing. We review ways to deliver the therapeutic genes or gene-regulatory elements to inner ear target cells using currently developed adeno-associated, virus (AAV)-derived vectors via clinical suitable delivery routes. We also discuss the various strategies used in gene therapy, such as gene addition, silencing, and editing.

## 2. Challenges and Advantages of Inner-Ear Anatomy for Cochlear Gene Therapy

**Figure 1:**
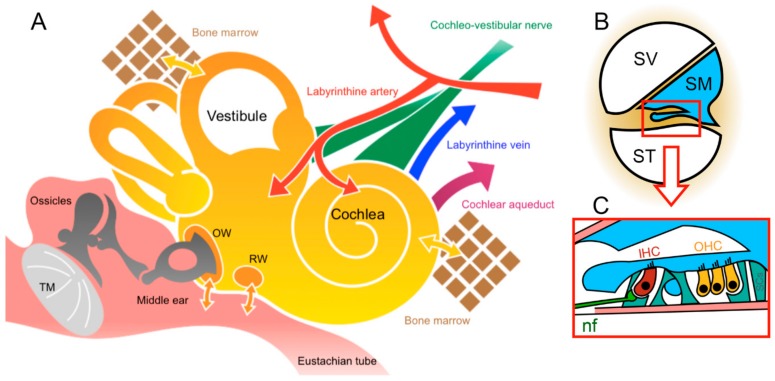
Inner ear anatomy and barriers. **A:** A schematic drawing of the structures of middle and inner ears, and inner-ear fluid flow and barriers. The tympanic membrane (TM) separates the external auditory canal from the middle ear that communicates with the nasopharynx via the Eustachian tube. The ossicular chain links the TM to the oval window (OW). Both round window (RW) and OW membranes form the connection between the middle ear and the cochlear perilymphatic space. The yellow arrows indicate communications between the perilymphatic spaces of the inner ear and the surrounding structures. **B:** Cross section of a single cochlear turn. The cochlea is made up of three canals: scala vestibuli (SV) and scala tympani (ST), filled with perilymph (in white), and scala media (SM), filled with endolymph (in blue). The red box indicates the organ of Corti. **C:** Shown is the organ of Corti. The organ of Corti located on the basilar membrane (in pink) is composed of mechanosensory cells, with three rows of outer hair cells (OHC) and one row of inner hair cells (IHC). Separating these hair cells are supporting cells (SCs). The nerve fibers (shown in green, nf) of the spiral ganglion neurons connect to sensory hair cells.

The inner ear is a complex, fluid-filled structure encapsulated in a very dense tissue (called otic capsule or bony labyrinth) of the temporal bone (Figure 1). The bony labyrinth of the inner ear houses a much smaller membranous labyrinth that is composed of two functional parts: the vestibular labyrinth and the cochlear labyrinth. The space located between the bony and membranous labyrinths is called perilymphatic space and filled with perilymph. The perilymphatic space communicates with cerebrospinal fluid (CSF) via the cochlear aqueduct and the cochlear modiolus in mammals (Figure 1), except in humans, where there is an individual variation in permeability between these two fluids (for a review of CSF and perilymph, see Reference [11]). The membranous labyrinth is filled with endolymph. The vestibular membranous labyrinth includes utricle, saccule, and three semicircular canals (anterior, lateral, and posterior semicircular canals) containing the receptors for the sense of equilibrium. The cochlear membranous labyrinth is called the cochlear duct (scala media) and houses the organ of Corti with its receptors for the sense of hearing (Figure 1).

Inner-ear gene therapy is challenging due to its inaccessible location and the blood-labyrinth barrier (i.e., the barrier between the vasculature and fluids of the inner ear, Figure 1). The blood-labyrinth barriers, including both blood-endolymph and blood-perilymph barriers, are represented by the tight junctions of the continuous endothelia of the inner-ear capillaries [12]. These barriers limit the entry into the inner ear fluids from the blood of compounds of high molecular weight, and of biomaterials [13], which makes cochlear gene therapy through a systemic delivery route challenging. Conversely, the cochlea is potentially an ideal organ for gene therapy. Its small volume necessitates only a limited amount of the virus. Furthermore, its relative isolation from other organ systems limits off-target effects to unwanted organs, and its liquid-filled structures facilitate viral delivery throughout the labyrinth. In addition, viral vectors applied to cerebrospinal fluid may enter the perilymph through the cochlear aqueduct (Figure 1) without a disturbance of hearing and balance structures.

Our knowledge of gene therapy is mostly gained through research in animals, especially in mouse models of human disease. Although many anatomic similarities exist between the mouse and the human inner ear, there are a number of differences, which may eventually influence the outcome of gene therapy. The discrepancy of the inner ear size in humans and in mice leads to different required volumes of viral vectors and a potential different spread of viral particles in the perilymphatic compartment.

## 3. Recombinant Adeno-Associated Virus Vectors

To date, a number of different viral vectors, including adenovirus (Ad), recombinant adeno-associated virus (rAAV), lentivirus, herpes simplex virus, and vaccinia virus have been developed [14,15]. Of these, rAAV has demonstrated the most potential for the development of clinical gene-therapy applications.

The rAAVs that enable to transduce a wide range of cells and tissues without detectable adverse effects is one of the safest strategies for gene therapy [16,17]. Moreover, they are non-pathogenic and non-replicative. From a translational point of view, their safety and efficiency have been proven in ocular gene therapy [18,19] and a large number of clinical trials [20]. Most rAAV vectors currently produced employ the AAV2 inverted terminal repeat sequences (ITRs) in their vector designs. Promoters commonly used include cytomegalovirus (CMV), elongation factor 1a (EF1a), simian virus 40 (SV40), and chicken β-actin (CBA) [21]. A major limitation of rAAV for gene therapy is its 4.7-kb limited packaging capacity of the expression cassette. Fortunately, several studies have demonstrated the success of dual-rAAV injection [22] using an overlapping vector strategy [8,9] and allowing the delivery of therapeutic genes up to 9 kb in size using rAAV vectors [23].

### 3.1. rAAV Trafficking and Transduction

rAAV follows several steps to achieve transgene expression. The first step requires binding to cell-surface receptors such as glycoproteins [24,25]. The sugar-binding preferences of different variants of AAV are dependent on their capsid sequence, which may impact the cell-type transduction preferences of different serotypes of AAV [26,27]. More recently, following an unbiased genetic screen, a universal AAV receptor, i.e., being used by a wide variety of different capsid serotypes, was discovered [28], which suggests that other determinants of AAV cellular entry should rather be considered to be co-receptors. Internalization by endocytosis is enhanced by interactions with these different co-receptors, depending on the AAV serotypes. These co-receptors also play a role in viral transduction and contribute to the cell and tissue selectivity of viral vectors. AAV2 uses αVβ5 integrins [29], fibroblast growth factor receptor 1 [30], hepatocyte growth factor receptor [31], αVβ1 integrin [32], and a laminin receptor [33].

The second step is to deliver single-stranded viral DNA to the host cell nucleus. For that, rAAV particles in endosomes must undergo a series of pH-dependent structural changes [34] and transport from the cytosol to the nucleus via the cytoskeleton network [35]. After endosomal escape, rAAV enters the nucleus via the nuclear pore complex [36,37], where it undergoes capsid uncoating to release the viral genome.

The third step is to convert single-stranded DNA into a double-stranded DNA. This conversion can be accomplished by second strand synthesis with host-cell DNA polymerases, or by strand annealing of the plus and minus strands, which leads to gene expression [38].

### 3.2. Approaches Used for Selective Tissue—Or Organ Targeting

To improve transduction efficiency and specificity, and modify tropism, several strategies have been developed such as pseudotyping, which uses the genome of one ITR serotype (commonly the ITR of serotype 2) with the capsid of another AAV serotype. This approach has allowed broad tissue tropisms [23]. Interestingly, synthetic AAV capsids have also been designed by reconstruction of ancestral sequences, and have allowed considerable improvements over the use of conventional rAAV vectors. To date, nine functional, ancestral AAVs have been generated [39]. Among them, Anc80L65 is a potent in vivo gene-therapy vector for targeting cochlear cells [40].

In addition to the pseudo-typing approach, another strategy is to use a specific promoter to restrict or enhance transgene expression in target cells or tissues. It has been reported that muscle creatine kinase and desmin promoters could also reach high levels of expression in skeletal muscle, but the myosin heavy-chain promoter may restrict transgene expression in cardiac muscle [21]. The use of the neuron-specific enolase promoter allowed neuron-specific expression [41,42]. A recent report also showed that local cochlear delivery of rAAV vectors with the CMV-beta-Globin hybrid promoter mainly drove transgene expression in cochlear sensory hair cells. By contrast, the CBA promoter was more efficient in supporting cells [43].

Lastly, more sophisticated strategies include the employment of small peptides or ligands inserted into the viral capsid or bispecific antibodies [44], and of biotin [45,46], that could interact with both the viral surface and the specific cell-surface receptor to achieve selective tissue or organ targeting.

### 3.3. AAV Immunogenicity and Strategies to Avoid

Traditionally, the cochlea is considered to be an immune-privilege organ. However, recent studies have shown specific resident immune cells in this sensory organ [47]. In addition, induced immune responses were seen in cochleae damaged by noise and ototoxic drugs [48,49,50]. Even though rAAV vectors showed relatively low immunogenicity compared to other virus-derived gene transfer vectors, such as recombinant adenovirus [51], an rAAV-induced immune reaction is also reported [52]. Immunity developed in response to natural AAV infection exists in humans [53], as well as in large animal models [54], which raises the issue of variable patient-dependent neutralization of therapeutic rAAV.

To avoid rAAV-induced immunogenicity in the cochlea, several strategies seem promising such as generated rAAV with a synthetic capsid presenting low antigenic similarity [55]. In addition, the route of delivery [56], the titer of viral vectors, which is closely related to the promoter being used [57,58] may have an impact on the immune response. It seems crucial to modulate rAAV-induced immunogenicity by using an undamaged local route with highly efficient low-dosing rAAV driving transgene expression under a specific promoter.

## 4. rAAV-Mediated Cochlear Gene Therapy

To date, several rAAV subtypes have been successfully used for the delivery of genetic material to different cochlear cells such as hair cells, support cells, and auditory nerve and spiral ligament, with little or no detectable damage to the organ of Corti [59,60]. rAAV-mediated cochlear gene therapies have been applied through cochlear local or systemic applications to several mouse models of genetic deafness with different degrees of success. However, although it should be noted that different subtypes of rAAV can be delivered locally, for the moment, only two can be used systemically (the synthetic AAV capsid Anc80L65, and the AAV9 capsid) because they can cross the blood-brain barrier and reach the cochlea after systemic injection [61].

### 4.1. Routes of Application

Although a systemic, intravenous route could be applied to deliver the genetic material to the cochlear cells, local delivery methods are more commonly used. Local delivery methods is supposed to cause fewer side-effects than systemic methods. In addition, local delivery ensures precise dosing and volume of viral solution injected into the inner ear.

#### 4.1.1. Systemic Route of Administration

**Figure 2:**
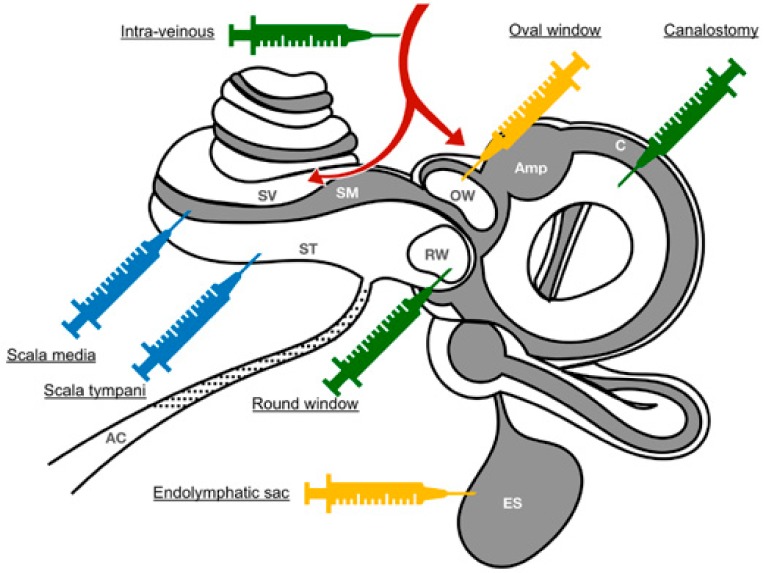
Schematic illustration of main administration routes tested in mice and potential suitable routes for human applications. The vectors can be delivered locally into the perilymph through the scala tympani (ST), trans-round-window (RW) membrane, or an oval-window (OW)/trans-stapedial injection into the endolymph through the scala media (SM) injection, canalostomy (C) or endolymphatic sac (ES) injection, and systemically through intravenous injection. The gray and white colors indicate the endolymphatic and perilymphatic spaces in the inner ear, respectively. The blue and green syringes indicate the main routes of administration tested in mice and the green and yellow syringes indicate the potential ones suitable for human applications. SV: scala vestibuli. Amp: ampulla.

Systemic injection of viral vectors is a recent technique developed to transduce the cochlear epithelium (Figure 2). Hudry et al. [61] showed that the synthetic AAV capsid Anc80L65, as well as the AAV9 capsid, can cross the blood-brain barrier and reach the cochlea after intravenous injection in post-natal and adult mice. Furthermore, Shibata et al. [62] showed that intravenous injection of rAAV9-eGFP gene resulted in binaural green fluorescent protein GFP expression in the inner hair cells (IHCs), spiral ganglion neurons (SGNs), and vestibular hair cells without hearing alteration in treated animals at postnatal day 30.

The advantage of systemic route is atraumatic and easy to carry out. The protocol in humans would simply require an intra-venous injection (Figure 2) under local anesthesia. In addition, systemic application may be useful to treat congenital syndromic and non-syndromic deafness before the alteration of the sensory epithelium begins. However, systemic delivery of the viral vectors to the cochlea faces numerous challenges, in particular avoiding adverse effects as a consequence of off-target binding to unwanted tissues or organs. This risk could be controlled by the use of a specific promoter. Other limitations of this route are the need for a high titer and a high volume of a viral vector. It is, therefore, more expensive than a local injection. While intravenous injections of rAAV2/9 can transduce the cochlear and vestibular sensorineural cells [62] in mice, the possibility of rAAV vectors crossing the blood-labyrinth barrier to deliver DNA to cochlear target cells in larger mammals must be confirmed before its potential clinical translation.

#### 4.1.2. Local Routes of Administration

Inner-ear gene therapies require a safe and effective route of administration that prevents damage to the delicate inner ear structures. Due to the inability of currently available viral vectors to spontaneously diffuse through the round-window membrane [63], intra-tympanic delivery is not possible. Thus, most of the local routes investigated to date focused on injection directly into the fluids of the inner ear. The local administration of therapeutic genetic materials into the cochlear perilymph may be through: (i) injection through the round-window membrane or into scala-tympani, (ii) the endolymph through a scala-media injection, and (iii) the perilymph and/or endolymph following canalostomy (see for review [14] and Figure 2). The canalostomic approach is believed to be promising for gene therapy in both cochlear and vestibular sense organs while preserving the hearing structures. The maximum volume allowed for injection corresponds to the volume of perilymph. A higher volume of injection in the perilymphatic compartment would lead in small mammals to overflow of the viral solution in the posterior fossa through the cochlear aqueduct (Figure 1). This maximum volume differs depending on the animal species studied including 0.62 µL in the mouse, 8.6 µL in the guinea pig, and 51 µL in humans [64].

##### Round Window Injection

To date, the common approach for rAAV delivery into the cochlea of adult or postnatal animals is the round window membrane (RWM) route. This surgical approach enables rapid and direct delivery into scala tympani. The round-window niche is easily accessible after opening the bulla by a post-auricular incision. Injection through the round-window membrane provides direct access to the perilymph of scala tympani (Figure 2). The viral vectors are, thus, in contact with the basilar membrane, from where they can pass to the endolymphatic space and reach the targeted cells. However, a transient elevation of hearing thresholds is often observed after opening the RWM and injecting viral vectors in adult mice [65,66]. Xia et al. [67] showed that opening the RWM followed by injection of viral vectors did not induce cochlear damage in neonatal mice. This route induces none or only transient vestibular disorders [55]. Some authors find a limited transgene expression at the apex of the cochlea after round window injection [68,69] possibly due to the preferential passage of the vector to the CSF via the cochlear aqueduct rather than along scala tympani (Figure 1).

Round window membrane injection is used clinically for cochlear implantation. Hearing loss secondary to perilymphatic leakage with RWM injection is a problem that can be avoided by plugging the RWM perforation with fascia [70,71]. A disadvantage of the RWM approach is that the distribution of the viral vector along the cochlear duct is challenging and, therefore, transduction tends to occur with a falling base-to-apex gradient in adult mice [71].

Promising studies indicate that this route may soon become feasible in humans. Dai et al. [72] showed that a peri-lymphatic injection via RWM of a phosphate-buffered saline vehicle at a volume sufficient for gene therapy delivery can be accomplished without causing permanent cochlea-vestibular alterations in Rhesus monkeys. György et al. [73] showed that round-window injection of viral vectors in non-human primates was technically feasible without causing cochlea-vestibular dysfunction. Using this route, they showed nearly complete transduction of inner hair cells, neurons, and supporting cells.

Recent increasing evidence supports the idea that the combination of cochlear implants with drug or gene therapy may reduce insertion trauma and preserve the residual hearing [74]. This combination treatment provides a promising perspective for future clinical translation.

##### Round-Window Membrane Diffusion

Instead of RWM perforation, an alternative method proposed is to facilitate the diffusion of viral vectors through the intact RWM following partial digestion by collagenase. Wang and Xia [67,75] showed good transduction of inner-ear cells after 10 min of partial digestion of the RWM without causing significant hearing loss in adult Guinea-pigs and 7-day postnatal mice.

This approach is likely the most promising route for clinical translation. This route is already used to treat several inner-ear diseases, including Meniere’s disease. Accessing the perilymphatic space through RWM diffusion is considered a generally low-risk procedure. Using an endoscope, it is now possible to directly enter into the round window niche via the external auditory canal in the majority of patients [76]. However, it is important to note that the real RWM is often obstructed by a pseudo-membrane in human temporal bones [77]. Therefore, in future translational studies focusing on cochlear viral-mediated gene administration, it is important to make sure that the entire RWM is in contact with a therapeutic solution after removal of the pseudo-membrane.

Another obstacle for RWM diffusion is the impermeability of this membrane for viral vectors. The RWM is a three-layered structure serving as a dynamic barrier to protect the inner ear. To overcome this obstacle, several strategies have been proposed to artificially increase transport across the RWM by partial digestion of RWM with collagenase [75], co-treatment with hyaluronic acid [70], or micro-perforations [78]. Future translational studies with larger mammals will teach us whether RWM diffusion is possible for rAAV-mediated cochlear gene therapies in humans.

##### Delivery via a Cochleostomy

Injection into the cochlear fluid spaces (scala tympani or scala medium) can be achieved through a hole drilled in the lateral wall of the cochlear basal turn (Figure 2). Injections into the perilymphatic space through a scala tympani cochleostomy may provide strong viral transduction with less cochlear damage than injection into the scala media (endolymphatic space). The scala media injection provides direct access for the vectors to the targeted cells. Administration of rAAV into the scala media resulted in a wide expression of a green fluorescent protein transgene within hair cells and supporting cells in guinea pigs, and, within the spiral ligament, Reissner’s membrane and SGNs in mice [59]. However, the opening of this very sensitive structure disturbs ionic homeostasis and is associated with cochlear damage if the injection is carried out at p5 in mice [59]. Viral injection into scala media is not possible in humans due to the risk of inner-ear injury and permanent sensorineural hearing loss.

##### Delivery via a Canalostomy

Canalostomy (Figure 2) is a relatively recent technique [59,65,79]. This route of delivery is technically as easy as the round-window route in adult and neonatal mice [80]. The posterior and lateral semicircular canals can be found in the post-auricular region after blunt dissection of the sternocleidomastoid muscle. Opening the canal allows insertion of the injection catheter (Figure 2). This technique does not require opening the bulla, and avoids disturbance of the middle ear during surgery. The viral vector crosses the vestibular organ to access the cochlea after canalostomic injection, which leads to good transduction of the utricular hair cells [40,73,81], sacular hair cells [40], supporting cells [81,82], and vestibular ganglion cells [40]. The cochlear epithelium is also transduced with a notably high level of transduction of apical hair cells [83]. Following intra-cochlear injection, the viral vector passes first through the scala vestibuli, which does not permit access to the endolymph, and to the helicotrema at the apex of the cochlea, where an endolymphatic passage is possible. The cells located in the apical portion of the cochlea must, therefore, be transduced first by the virus. The injection site via canalostomy is, thus, remote to the cochlea, and this prevents damage to cochlear cells and prevents potential hearing loss [40,79,81].

As a limitation of this route of administration, the space into which the vector is injected remains uncertain. The membranous labyrinth in the semicircular canals adheres to the bony canal along its convex wall [84]. The opening of the bony canal is accompanied by a high risk of opening the membranous labyrinth, and the injection may be made into the endolymphatic space. In addition, the canal may be post-operatively obstructed by fibrotic tissues, which raises the question of inflammation and permanent lesions of the vestibular epithelium. During the first days following surgery, a transient vestibular disorder is often reported. However, Suzuki et al. [40] reported no modification of vestibular evoked potentials observed two weeks after a viral injection. These results suggest that, despite fibrosis of the canal, no permanent vestibular damage occurs.

Canalostomy is a potentially suitable route for human gene therapy. This surgical approach is similar to that of cochlear implant surgery. This route would have the same benefits in humans as in mice. Nevertheless, it may cause fibrosis of the canal and vestibular disorders. Even if in mice the position of the canula is controversial due to the small size of the canal itself (around 100 um [79]), the relatively larger structures of the human inner ear (around 1 mm [85]) would likely allow a better discrimination of perilymphatic space from the endolymphatic space. The control of the depth and the strength of the insertion of the canula into the canal would, thus, help avoid lesions of the membranous labyrinth and possibly fibrosis. Robotic surgery could be a way to do this safely. The question of the closure of the hole made in the canal is crucial, too, assuming that a “third-window syndrome” could occur if a defect in the integrity of the bony structure remains.

##### Delivery via a Combined Approach

Yoshimura and colleagues [71] proposed an approach combining the canalostomy and a round window injection. This method allows the preferential perfusion flow from the base to the apex of the cochlea. This promising new method permits a near-complete transduction of targeted cells without affecting auditory function. Moreover, the double opening of the perilymphatic space allows relief of any pressure possibly applied on the sensory epithelium during the injection. Cochlear implants work by direct electrical stimulation of the residual auditory nerve in the deafened cochlea, which allows for a restoration hearing in patients. Another promising approach for rAAV-based therapeutic gene products delivery is to coat the electrodes of cochlear implants with viral vectors to protect residual hearing [86,87].

##### Oval-Window/Trans-Stapedial Injection

Unlike the round window, the oval window is not covered by a membrane, but by the footplate of the stapes. In mice, the stapedial artery courses across the footplate, and injection by this route carries a lethal risk for the animal in case of injury, which makes it inappropriate [11]. However, in humans, the stapedial artery is not present, and the injection through the footplate of the stapes seems technically feasible. The trans-stapedial route (Figure 2), therefore, appears as relevant as the round-window route. The surgical approach is a routine, well-established technique, consisting of a laser stapedotomy, as in otosclerosis surgery. Dai et al. showed no significant auditory or vestibular disfunction using this route for rAAV delivery in Rhesus monkeys [72]. However, the authors pointed out the limits of this technique, particularly some visible reflux of fluid around the injection catheter, which makes the total amount of volume injected uncertain. Technical improvement could, thus, be required before its clinical translation.

##### Endolymphatic sac Injection

The endolymphatic sac lies in the endocranial side of the petrous bone. It is connected to the endolymphatic compartment by the endolymphatic duct [88]. Surgery of the endolymphatic sac is proposed in some cases of treatment-resistant Meniere’s disease. The surgical incision of the sac is not associated with auditory or balance disfunction [89], which is required for a rescue therapeutic strategy. Both anatomical findings and physiological measurements suggest that the endolymphatic duct acts as a mechanical valve, which allows the flow of endolymph from the sac to the endolymphatic compartment [90]. Injection into the sac after a surgical approach is, thus, promising (Figure 2). The viral vectors would, thus, be safely injected into the endolyphatic compartment and should spontaneously directly reach the targeted cells. However, the surgical approach consists of a mastoidectomy with a large decompression of the posterior fossa plate and is, thus, accompanied by a greater surgical risk than the other routes.

### 4.2. Preclinical Targets

rAAV provides a long-lasting transgene expression in non-dividing cells, and the small size of the virion (about 20 nm) favors a good diffusion across cellular barriers. These two capacities allow it to reach cochlear cells and ensure transgene expression in the different cochlear cell types, e.g., cochlear hair cells, supporting cells, auditory nerve, and spiral ligament with little, if any, alteration of the cochlear function [60]. These benefits of rAAV make it a significant new approach for gene addition, gene suppression (RNAi), gene editing, and treatment or prevention of genetic deafness.

#### 4.2.1. Gene Addition

The promise of rAAV gene therapy has been achieved in particular regarding rAAV delivery of wild-type genes to address mutant or absent genes in experimental and clinical settings. In this case, we summarize some successful results of rAAV-mediated expression of exogenous wild-type genes needed for inner ear function for treating congenital or early-onset hearing loss (Table 1).

The mutation in *TMC1* gene encoding the transmembrane, channel–like protein isoforms 1 (TMC1) may cause autosomal recessive (DFNB7/11) or dominant (DFNA36) deafness. Beethoven (Bth)-mouse models carrying either a targeted deletion of *Tmc1* or a dominant *Tmc1* point mutation are good models for human DFNB7/11 or DFNA36 conditions [91]. Askew et al. [92] showed that delivery of rAAV2/1, carrying either the wild type *Tmc1* or *Tmc2* gene through round-window membrane administration in deaf mice lacking TMC1 or TMC2, partially restored sensory transduction and hearing. Furthermore, *Tmc2* gene therapy induced the partial recovery of auditory brainstem responses (ABRs). However, without improvement in the startle response in mice bearing dominant Bth mutations in the *Tmc1* gene, indicating the addition of exogenous wild-type *Tmc2* gene may be insufficient to overcome the dominant Bth mutation in a behaviorally relevant assay.

The *USH1C* gene encodes the harmonin protein, which plays a critical role in hair cell bundle formation, structure, and function. The USH1C.216G>A (216A) mutation is the main cause of Usher 1 syndrome [93]. This mutation creates a cryptic 5′ splice site, which results in a frameshift and truncated harmonin protein [94] leading to an alteration in the expression of all conventional harmonin isoforms [93]. Homozygous *Ush1c c.216G>A* mice (c.216AA) displayed severe hearing loss, with disorganized hair bundles and loss of IHCs and outer hair cells (OHCs) in the middle and basal turns of the cochlea by one month of age [93]. Pan et al. [10] showed that injection of rAAV2/Anc80L65, driving the harmonin-a1 or harmonin-b1 genes via the RWM route in early postnatal mice, successfully transduced large numbers of IHCs, OHCs, and vestibular cells and rescued harmonin expression and localization, and, thus, successfully restored hearing and balance in *Ush1c* mice.

Isgrig et al. [95] showed that injection of rAAV2/8, driving wild-type whirlin through unilateral, posterior semicircular-canal route in neonatal deaf whirler mice, a model of Usher syndrome type 1G was able to restore their balance and hearing function. Lastly, the intracochlear injection of rAAV2/8, driving cDNA encoding the scaffold protein SENS in a newborn mouse model of Usher syndrome type 1G effectively restored the structure and function of inner-ear hair cells, and rescued the balance and low frequency hearing [96].

Otoferlin protein plays a key role in the glutamate release in the IHCs synapse. Mutation in the *OTOF* gene causes profound autosomal recessive hearing loss (DFNB9) [97]. Al-Moyed et al. showed that correct reassembly of the full-length otoferlin cDNA in IHCs by a dual-rAAV strategy restores exocytosis and partially rescues auditory function in neo-natal *Otof^−/−^* mice [8]. Using the same dual-rAAV strategy, Akil et al. found total hearing recovery in both treated neo-natal mice and young adult (P30) mice [9]. This finding highlights the ability of local gene therapy to rescue hearing even in young adult mice, which raises hope for future post-natal gene therapy trials in DFNB9 patients.

Lastly, the vesicular glutamate transporter VGLUT3 plays an important role in concentrating glutamate into synaptic vesicles of the sensory IHCs before it is released onto receptors of auditory-nerve terminals. Mutations in the *SLC17A8* gene encoding VGLUT3 causes autosomal dominant deafness in humans. VGLUT3-deficient mice lacked ABRs to acoustic stimuli, even though auditory brainstem responses (ABRs) could be elicited by electrical stimuli [98]. A successful restoration of hearing was demonstrated in this *Slc17a8* knockout mouse model by reinstating the expression of *Slc17a8* via postnatal rAAV-mediated delivery, as shown by the restoration of synaptic transmission and hearing [99].

These exciting results with rAAV-mediated gene addition therapies provide a promising perspective for future clinical cochlear gene therapy to address defective genes responsible for inner ear diseases. However, it should be noted that, in gene addition strategy, the mutated gene remains and this sometime leads to dominant effects impeding therapy efficiency. To address these limitations of gene addition strategy, different strategies have emerged, which range from mutated gene suppression and replacement to specific gene or RNA editing.

#### 4.2.2. RNAi

The RNA interference (RNAi) pathway can be diverted for the downregulation of gene expression for genetic conditions in which the mutation leads to a toxic gain of function. RNAi presents some advantages over modern gene editing techniques such as clustered regularly interspersed palindromic repeats (CRISPR)/Cas9 nuclease. First, this technique does not induce an immune reaction, unlike Cas9 protein [104]. Second, the RNAi is proven to work efficiently in most mammalian cell types. Third, RNAi can theoretically target any protein-coding gene. Lastly, the suppression of the target mRNA can be stable if using a viral vector for delivery [105]. The limitations of RNAi are that this technique cannot induce activation of a gene, or correction of a mutation, and can only suppress post-transcriptionally the targeted gene. It is associated with off-target effects, and, as yet, there is no methodology to fully avoid them [106].

In the inner ear, this strategy has been successfully used to rescue genetic deafness (Table 1). Inner-ear sensory epithelial development, function, and repair are all dependent on the intercellular communication of the gap junction [107]. Mutations in the connexin *GJB2* and *GJB6* genes, encoding respectively CX26 and CX30, cause syndromic and non-syndromic deafness [107]. Maeda and colleagues [100] showed that allele-specific silencing of the dominant disease allele (R75W) of the *GJB2* gene encoding Gap junction protein beta2 with the RNAi method rescued hearing in mice that would otherwise become deaf after round-window injection of liposomes delivering a mutated sequence in the *GJB2* gene. These results represent a very encouraging breakthrough since connexin mutations cause the majority of genetic deafness.

#### 4.2.3. Antisense Oligonucleotide

Antisense oligonucleotides (ASO) belong to the same family of antisense molecules as shRNA and miRNA. These linear single stranded DNA (ssDNA) recruit intracellular enzymes such as RNAses, which cleave targeted-RNA. ASO present the advantage of being easy to produce and to modify. However, they have the potential of binding proteins as well and have a risk of off-targets side-effects [108], and, contrary to viral vector-mediated gene therapy, they need to be administered on a regular basis for a long-term effect.

Lentz et al. [101] successfully treated a mouse model of type 1 Usher, which harbors the human 216A mutation, by systemic injections of an ASO. This promising result is mitigated by the necessity of a treatment before the end of the maturation of the cochlea, so it would require intrauterine injections in humans (Table 1). Nevertheless, this study represents a strong proof of concept that ASO can be used for cochlear gene therapy.

#### 4.2.4. Gene Editing

The development of genome-editing technologies has revolutionized the field of genomic medicine. These technologies allow correction of mutations that cause disease, addition of therapeutic genes to specific sites in the genome, and deletion of the deleterious genes or genome sequences. In this case, we summarize gene editing using the clustered regularly interspersed palindromic repeats (CRISPR)/CRISPR associated protein 9 (Cas9) system. This technique was initially adapted to introduce stable insertions and deletions in a target sequence. However, if the deletion lead to disruption of the codon-reading frame, it can induce disruption of the transcription of the gene, and, thus, repression of gene expression [109]. In addition, CRISPRi (association of transcription repression domain) or CRISPRa (association with transcription activation domain) can repress or activate a target gene [110].

The CRISPR-CAS nuclease system works with two molecules: a synthetic, short-guide RNA serving to find and bind to a specific sequence in the DNA, and a Cas-like enzyme, which produces double-stranded breaks in the host DNA in a specific manner. Following the cut, DNA double-stranded breaks can be repaired either by non-homologous end joining (NHEJ) or homology-directed repair (HDR). The error-prone NHEJ mechanism leads to sequence insertion or deletion, which allows knockout of the target gene. HDR, on the other hand, performs a precise DNA repair using a dsDNA template provided with the short-guide RNA and a Cas-like enzyme. Therefore, this provides optional methods for the correction of genetic disorders [111,112]. rAAVs have mostly been the vector of choice for CRISPR genome editing [113].

The CRISPR-Cas9 system has already been tested with a nucleofection technique in patient-derived hair-cell-like cells differentiated from iPSCs of deaf patients bearing MYO7A or MYO15A mutations, and demonstrated the successful correction of these mutations as well as morphological and functional restoration of hiPSC [114]. A recent pioneering study showed that neonatal, scala-media delivery of cationic, lipid-mediated CRISPR–Cas9 complexes in the ‘Beethoven’ mouse model successfully corrected mutant alleles, improved hair-cell survival, and restored hearing [102]. More recently, the same group showed that a protospacer-adjacent motif variant of Staphylococcus aureus Cas9 (SaCas9-KKH) can selectively and efficiently disrupt the mutant allele, but not the wild-type Tmc1/TMC1 allele in Beethoven mice. By using rAAV-mediated SaCas9-KKH delivery in postnatal day 1 Beethoven mice, they were able to prevent deafness efficiently for up to one year after transduction [103] (Table 1). These works convincingly demonstrated the possibility of using newly developed genome-editing strategies to restore genetically-produced hearing loss in deafened patients carrying monogenic mutations.

### 4.3. Risk of Getting Side-Effects

An important point that needs to be considered is the risk of off-target side effects. Even though cochlear gene therapy is often considered a promising site for viral therapies due to the organ’s relatively isolated nature, we must take into consideration the communications between the perilymph and the CSF through a cochlear aqueduct, modiulus, and bone marrow (Figure 1) [115]. In fact, transduction of cerebellum and contralateral ear is often reported after perilymphatic injection of viral vectors [79], and the level of brain and contralateral ear transduction correlated with the injected volume. Strong brain and contralateral ear transduction were particularly reported in the early studies by using a protocol of eight days of perfusion of about 100 µL of viral vectors [116]. However, no contralateral transduction was found after a single-injection of 1 to 2 µL [83]. The route of administration also has an impact on the passage of the CSF. The further the injection site from the cochlear aqueduct is, the weaker the passage is.

Thalen et al. [117] demonstrated that the efflux observed when the otic capsule is experimentally perforated is due to the entry of CSF through the cochlear aqueduct. It is, therefore, logical that excess pressure in the perilymphatic space following experimental injection induces a backward perilymph flow to the posterior fossa.

In rodents, the most significant communication route seems to be the cochlear aqueduct. Modiulus passage is, however, also possible. The histology of the vestibular and cochlear nerve shows no tight border between perilymph and the bottom of the inner auditory canal [118]. Kho et al. first showed the diffusion to the bone marrow of viral vectors. They detected the transgene in the temporal bone marrow of the ipsilateral and the contralateral ear after a single perilymphatic injection of rAAV. Few other authors reported bone marrow transduction, but this transduction was, however, rarely sought. It is noteworthy that all these studies used a ubiquitous promoter to direct the level of expression of the transgene.

In addition to these proofs of transduction of local regional tissues surrounding the inner ear, Landegger [55] showed a humoral response to the viral capsid following the injection of synthetic rAAV. We can, thus, assume that immunological reactions can be seen in humans, which raises the question of a decline in gene-transfer efficacy over time [119].

In humans, a communication between the inner ear and CSF also exists. The cochlear aqueduct is less widely open, as suggested in a study of 101 temporal bones. Only 34% of cochlear aqueducts were fully open while others presented their central lumen filled with connective tissues or bone [120]. The free passage of fluid along the cochlear aqueduct is still controversial [121]. Nakashima et al. [122] showed in 2012 that Gadolinium moves into the CSF via the internal auditory meatus. Pathological situations inform us of the possibility of passage for viral and bacterial pathogens, and even erythrocytes between the inner ear and CSF in both directions [123,124]. This passage is much more common in infants possibly because of the shorter length of the cochlear aqueduct [125].

These data highlight the risk in humans of potential off-target transduction and humoral responses after local perilymphatic injection of viral vectors.

## 5. Future Directions

In this review, we have provided a rAAV-centric view of current trends and challenges in the field of cochlear gene therapy. As technology continues to advance, the field of rAAV-based gene-therapy strategies may become so diverse, and accelerate so rapidly, that some of these technologies may fall out of favor before reaching the clinic, while others may translate to clinical practice.

Considerable progress has been made in the development of gene-delivery vector systems. Among all the vectors developed to date, rAAV may have the greatest prospects for transition to clinical trials. Today, serotypes 8, 9, and Anc80L65 have proven their efficiency in transducing IHC, OHC, support cells, and neurons. However, the efficiency and specificity of the gene-delivery agent needs to continue to improve. The capsid protein-engineering can ameliorate viral tropism, so that viruses can be regulated to preferentially target a subpopulation of the cochlear cells, while minimizing their off-target effects. The incorporation of promoters specific to the cell type will allow precise transgene expression in desired cell types within the cochlea. The current exponential growth of clinical trials using rAAV vectors suggests that we are only at the beginning of what is achievable for a harmless virus that has now become a programmable vector for improving human health.

Considering safety, efficacy, and easy operation in clinical practice, the intact RWM may be the most promising approach for gene delivery to the inner ear. The transfection efficiency with this approach could be further increased after developing agents enhancing the permeability of RWM to viral vectors. Alternately, canalostomy could be another suitable route for clinical inner ear gene delivery. However, for efficient treatments, one should take into account associated risks to the hearing and vestibular systems. Studies are, thus, required to ensure that injection into the human cochlea will not be harmful. Off-target side effects also have to be considered when manipulating genetic therapies near the brain, and with demonstrated risks of systemic passage.

In the near future, a combination of microsurgical and robotic tools with classic otological surgery could make therapeutic interventions more precise, and less traumatic during infusion of gene-delivery vectors. In addition, surgical innovations and technologies could allow the monitoring of cochlear electrophysiological changes, which minimizes the risk of cochlear damage in real time during therapeutic interventions [126].

As for the discovery of the pathogenic mechanisms of genetic deafness, gene therapy for hearing loss could become personalized. It may involve not only gene replacement by effective viral transduction, but also the deactivation of a dominant negative allele using miRNA or shRNA. The off-target effects will be decreased by improving the specificity of the therapy. CRISPR-Cas systems will be used in the near future for precise DNA or RNA editing for each patient.

Currently, a three-part, multi-center, open-label, single-dose study aimed at assessing the safety, tolerability, and efficacy of intra-labyrinthine administration of a recombinant adenovirus 5 vector containing the human atonal transcription factor cDNA (CGF166) in patients with severe-to-profound hearing loss is ongoing in the United States (Clinicaltrials.gov Identifier: NCT02132130). Another clinical in vitro study is aiming at investigating viral transduction of rAAV in human inner-ear cells that were collected during non-conservative surgeries for vestibular schwannoma (Clinicaltrials.gov Identifier: NCT03996824).

For the successful clinical translation of gene therapy for treating or preventing inner ear genetic diseases, important questions such as off-target side effects, biodistribution of vector components, and the risk of carcinogenesis remain to be addressed through preclinical trials used in non-human primates or humanized models such as: (i) explants of human adult cochlear epithelium from surgical resection of the cochlea or from donors in the state of brain death, and (ii) inner-ear organoids generated from human-derived iPSCs [65,66] or from deaf patients. The generation of in vitro pathological models for each genetically deaf patient would allow transitions into personalized and precise medicine.

## Figures and Tables

**Table 1:** **Cochlear gene therapies**. Summarizing of the recent proof-of-principle studies demonstrating the therapeutic potential of gene therapies for preventing or treating inner ear genetic diseases. ABR: auditory brainstem response. RWM: round-window membrane. IHC: inner hair cell. ASO: antisense oligonucleotides. CBA: chicken β-actin promotor. CMV: cytomegalovirus promotor.

Deafness	Mouse Models	Therapeutic Strategies	Vectors and Promotors	Routes	Outcomes	Reference
DFNB7/11 and DFNA36 Usher 1C syndrome Usher IG syndrome DFNB9 DFNA25	*Tmc1*^−/−^, *Tmc2*^−/−^, *Tmc1/2*^−/−^, *Tmc1*-Bth *Ush1c c.216G > A* Ush1g^−^^/^^−^ Otof^−^^/^^−^ *Slc17a8 ^−/−^*	*Tmc1 or Tmc2* gene addition harmonin-a1 or harmonin-b1 gene addition cDNA SENS addition Otoferlin cDNA addition VGLU3 cDNA addition	rAAV2/1-CBA rAAV2/Anc80L65-CMV rAAV2/8-CBA Dual rAAV-CBA Dual rAAV2/6-CBA/CMV AAV1-CBA	RWM P0-P2 RWM P1 RWM P2 RWM P10-P30 RWM P6-P7 RWM P10 or cochleostomy	Partially restored sensory transduction, ABR, and acoustic startle reflexes. Restoration of hearing and balance. Rescue of balance and low frequency hearing. Complete hearing restoration Partial restoration of IHC exocytosis and hearing. Complete restoration of ABR thresholds, partial rescue of the startle response	[92] [10] [95,96] [9] [8] [99]
Cx26 deafness Usher 1C syndrome	GJB2 _R75W_ *USH1C* ^216^ Knock-In	siRNA against disease allele (R75W) ASO to block 216A cryptic splicing	Liposome complex	RWM in adult Intraperitoneal Injection P3-P16 or adult	Partial restoration of hearing Partially rescued vestibular function and hearing	[100] [101]
DFNA36	*Tmc1*-Bth	CRISPR-Cas9 Gene Editing	Cationic lipid or rAAV2/Anc80L65-CMV	Scala media injection P1	Effective prevention of deafness	[102,103]
